# An Environmentally Sustainable Software-Defined Networking Data Dissemination Method for Mixed Traffic Flows in RSU Clouds with Energy Restriction

**DOI:** 10.3390/ijerph192215112

**Published:** 2022-11-16

**Authors:** Hongming Li, Dongxiu Ou, Yuqing Ji

**Affiliations:** 1 Key Laboratory of Road and Traffic Engineering, Ministry of Education, School of Transportation Engineering, Tongji University, Shanghai 201804, China; 2 Key Laboratory of Railway Industry of Proactive Safety and Risk Control, School of Transportation Engineering, Tongji University, Shanghai 201804, China

**Keywords:** mobile edge computing (MEC), energy restriction, intelligent transportation systems (ITS), energy consumptions, cloud resource management (CRM), software-defined networks (SDN), environmental sustainability

## Abstract

The connected multi road side unit (RSU) environment can be envisioned as the RSU cloud. In this paper, the Software-Defined Networking (SDN) framework is utilized to dynamically reconfigure the RSU clouds for the mixed traffic flows with energy restrictions, which are composed of five categories of vehicles with distinctive communication demands. An environmentally sustainable SDN data dissemination method for safer and greener transportation solutions is thus proposed, aiming to achieve the lowest overall SDN cloud delay with the least working hosts and minimum energy consumption, which is a mixed integer linear programming problem (MILP). To solve the problem, Joint optimization algorithms with Finite resources (JF) in three hyperparameters versions, JF (DW = 0.3, HW = 0.7), JF (DW = 0.5, HW = 0.5) and JF (DW = 0.7, HW = 0.3), were proposed, which are in contrast with single-objective optimization algorithms, the Host Optimization (H) algorithm, and the Delay optimization (D) algorithm. Results show that JF (DW = 0.3, HW = 0.7) and JF (DW = 0.5, HW = 0.5), when compared with the D algorithm, usually had slightly larger cloud delays, but fewer working hosts and energy consumptions, which has vital significance for enhancing energy efficiency and environmental protection, and shows the superiority of JFs over the D algorithm. Meanwhile, the H algorithm had the least working hosts and fewest energy consumptions under the same conditions, but completely ignored the explosive surge of delay, which is not desirable for most cases of the SDN RSU cloud. Further analysis showed that the larger the network topology of the SDN cloud, the harder it was to find a feasible network configuration. Therefore, when designing an environmentally sustainable SDN RSU cloud for the greener future mobility of intelligent transportation systems, its size should be limited or partitioned into a relatively small topology.

## 1. Introduction

China plays a leading role in global green development as the largest developing economy, and it is essential to manage the balance between the economy and protection of environmental resources with energy restriction [1]. Furthermore, energy consumption is of great importance when it comes to maintaining the environmental sustainability of the planet and ecosystem. China has set targets to curb carbon emissions by 2030 and become carbon neutral by 2060, in which case energy conservation is vital for achieving reduced carbon emissions [2]. In the process of implementing the actual emission target, the different drivers of carbon emissions are complex and variable because of the huge differences in regional economic development, industrial structure, and energy structure [3]. Meanwhile, transportation is an important cornerstone of economic development for a country, and intelligent transportation systems (ITS) could build a sustainable and favorable environment for the mixed traffic flows, which consist of normal vehicles, trams, and intelligent connected vehicles in the open and mixed road environment. Furthermore, traffic congestion plays an important role in transportation, which is occurring in almost all core cities all over the world [4]. ITS will be of great importance in improving the efficiency and safety of the transportation operation. Time is one of the most important attributes in transport-related research [5], but traffic congestion could cause a great waste of time for the transportation system. The SDN framework-supported RSU cloud for greener future mobility of the ITS, which consists of connected multi-RSU environments, can be used for traffic congestion mitigation. Mobile Edge Computing (MEC) for optimizing vehicle pathways can be supported by the SDN RSU Cloud. The real-time data is essential for providing the safety environment for the vehicles, planes, and ships, and the information from sensor data about timestamps, geographical positions, and ship heading plays a critical role in retrieving weather conditions [6]. While ensuring the sustainable and smooth operation of transportation systems, traffic safety is another urgent problem that cannot be ignored. Several safety systems have been designed to improve traffic safety, and their benefits may be reduced, due to bad risk compensation or system misuse [7]. For ITS, the safety applications enabled by the SDN RSU cloud can be used in the shared mobility services in connected and automated environments to improve safety.

In this article, for the mixed traffic flow on the road, where vehicles are divided into five categories, both normal vehicles and autonomous vehicles are separated into two cases with or without MEC assistance, as well as the trams with MEC enabled. The connected and autonomous vehicles (CAVs), with their capabilities of real-time communication and precise motion control, can provide great potential to facilitate all kinds of intelligent transportation system applications [8]. In addition, a variety of ITS applications for the mixed traffic flow will work in vehicle ad-hoc networks (VANETs) with the SDN RSU cloud enabled [9]. It is supposed that the application scenario of the intelligent transportation system is located in a typical urban area. Therefore, modeling the communication demands of the mixed traffic flow is an urgent issue, and to the best of our knowledge, few works are trying to separate the vehicles into different categories for modeling the communication demands in detail. After the detailed modeling of mixed traffic flow communication demands, then the fundamental component of the proposed data dissemination approach in the RSUs cloud is load balancing. When the demands are unevenly distributed in the Vehicle-to-Vehicle (V2V) and Vehicle-to-Infrastructure (V2I) networks, it is then time to make the loads in the cloud balanced. Load balancing procedures, in which responsibility is shared by increasing the number of participating RSUs to transfer the overload of existing RSUs to other RSUs, are important for the effective dissemination of data in the RSU cloud networks [10]. Therefore, achieving effective communication for the internet of the various vehicles, while making maximum use of the limited network resources of the SDN RSU cloud, is a knotty problem. Ali et al. have proposed an enhanced cooperative load balancing approach for efficient data dissemination in vehicle ad-hoc networks, yet do not take the energy consumption into consideration [10]. Another work by Hao Y et al. has been conducted on the resource allocation for V2V communications, which used the deep reinforcement learning-based approach [11]; still, they did not consider the energy restriction issues. In this article, the energy restriction has been considered for a data dissemination approach under the SDN framework. Thus, an environmentally sustainable software-defined networking data dissemination method for mixed traffic flows in RSU clouds with energy restriction has been proposed.

The structure of the article is organized as follows: In Section 2, the related works describe the contributions of predecessors and present the data dissemination problem in detail. In Section 3, we first introduce the ITS application scenario, and then model the communication demands of the mixed traffic flow. Furthermore, we illustrate how RSU plays three different roles—the OpenFlow Controller, the RSU Cloud Resource Manager and the normal RSU function—to establish the mechanism of the SDN RSU cloud. Moreover, we design an SDN data dissemination method with energy restriction, whose goal is to achieve the overall minimum SDN cloud delay with the least number of hosts and energy consumptions, which can be summarized as the mixed integer linear programming problem (MILP). In Section 4, the experiments first introduce the Joint optimization algorithms with Finite resources (JF) in three hyperparameter versions, which are in contrast with single-objective optimization algorithms, the Host Optimization (H) algorithm, and the Delay optimization (D) algorithm. Section 4 then depicts a network topology consisting of 12 RSUs, whose experimental environments and initial network configurations are presented in detail. What is more, in Section 4, the analysis for the service number, cloud delay, the number of working hosts, and energy consumptions are all conducted. Section 5 implements further and detailed results analysis. Section 6 concludes this article.

## 2. Related Work

Many scientists have devoted a great deal of effort to data dissemination in V2I and V2V communication research. Some studies have focused on the communications between vehicles in only one regional RSU. A framework supported by RSUs within which adjustable methods of data dissemination can be used has been proposed by Liu et al. [12]. Zhang et al. assessed the impact of upload requirements on data quality, and proposed a two-step plan to balance the download and update requirements [13].

On the one hand, since the traditional load transfer mechanism could no longer meet the requirements of VANETs, achieving the efficient communication of vehicles in a multi-RSU environment has been studied. Wu et al. pre-established access points and adjusted the number of access points (APs) according to the types of traffic; they proposed an access point controllers-based scheme, QualityScan, that regularly collects the state of multiple RSUs (APs) to predict network traffic at the next moment [14]. Lochert et al. studied the dissemination of information in VANETs under urban scenarios and further compared the information dissemination effectiveness with and without RSUs; they concluded that a multi-connected RSU environment can significantly improve communication performance [15]. Shahverdy et al. proposed a scheduling algorithm based on multi-RSU architecture to deploy multi-RSU on high-speed backbone connections; each RSU executed two separate queues: (1) Download the request from the start; (2) Resume download [16]. Wang et al. proposed a vehicle-to-infrastructure (V2I) and a vehicle-to-vehicle (V2V) joint communication system through estimation algorithms; they recommended that V2I be used to broadcast information to vehicles within the RSU, and then V2V be used to transmit information to vehicles outside the RSU [17].

On the other hand, Cyber Physics Systems (CPS), with the help of MEC, are working on a solution to the precarious and long-awaited connection between the device and the cloud data center, in which mixed traffic flow vehicles can be considered as CPS devices. Gu et al. [18] investigated three factors related to base station association, task allocation, and virtual machine configuration to achieve cost-effective medical CPS (FC-MCPS). Behbehani et al. have designed a vehicle cloud architecture of edge computing nodes and central clouds to distribute computing needs evenly while minimizing power consumption [19]. More importantly, He et al. [20] proposed an integrated framework for car networking, caching, and computing, and a large number of parameters in the model were calculated using a deep reinforcement learning approach. Additionally, Zhu et al. [21] proposed a new multi-modal deep reinforcement learning method (RL) based on a hybrid strategy for mobile edge computing; the corresponding online algorithm can theoretically guarantee the convergence of learning.

However, unlike traditional V2I and V2V studies, software-defined networks are utilized in our study. After decades of development of the traditional network structure, the emergence of new network requirements has brought challenges to the network. Therefore, SDN is set up to separate control and forwarding functions, to logically centralize control functions, and to open and program the network. In 2008, McKeown et al. published the OpenFlow protocol, which was the first communication interface between control and data planes, and is presently the most popular SDN southbound interface protocol [22]. The OpenFlow protocol is the most widely used protocol in SDN, and it has almost become the actual standard for SDN southbound interfaces. OpenFlow Controller, OpenFlow Switch, and OpenFlow Protocol are three important aspects of OpenFlow Components, and are used to improve bandwidth utilization in wired networks. At the same time, Google has successfully applied software-defined networks to its data network [23]. In this paper, the communication requirements of mixed traffic flow are modeled, load balancing is considered to reduce the overall workload of the system, and cloud resource management for the SDN RSU cloud is carried out. The application of intelligent transportation system tries to meet the changing demand by defining network configuration as the number of service hosts and effective data forwarding rules, while cloud resource management looks to find the best network configuration through the computation and optimal configuration of network resources. The proposed SDN data dissemination method for safer and greener transportation solutions looks to find the optimal network configuration so that the network can achieve the minimum SDN cloud latency with the minimum number of hosts and energy consumptions.

## 3. Materials and Methodology

### 3.1. Mixed Traffic Flow

Mobile edge computing (MEC) works with the SDN RSU cloud to reduce any lag time on the ITS applications on auto pilot. At present, for autonomous vehicles (with MEC), a large number of them have high-quality requirements for computing capability, such as virtual reality (VR), augmented reality (AR), high-definition live broadcast, video surveillance, etc. All of these services are extremely sensitive to bandwidth and delay. There are various types of ITS applications, that is, the communication demands are summed up according to the categories of information from the five types of vehicles in the mixed traffic flow.

**Figure 1:**
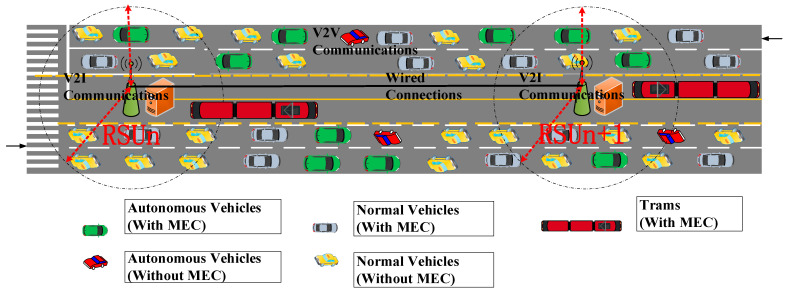
Vehicle communication demands of the mixed traffic flow.

In this study, we hypothesize that the application scenario of intelligent transportation systems is located in typical urban areas. In a typical urban scenario, the RSUs are located in the middle of the road, and mixed traffic flows consist of five categories of vehicles, as shown in Figure 1.

From what can be seen in Figure 1, there are five categories of vehicles, which are autonomous vehicles (with MEC), autonomous vehicles (without MEC), normal vehicles (with MEC), normal vehicles (without MEC), and trams (with MEC). It is worth noting that the trams can appear in the coverage area of some RSUs, while other RSUs do not cover them, since a limited number of trams operate on the track at a slow speed. For the RSUs as shown in Figure 1, all the RSUs are wired and connected with a high-speed ethernet backbone. For the vehicles in the coverage area of the RSU, they are communicating with the RSUs using V2I communications, while the vehicles outside the coverage area of the RSU relay information using V2V communications.

### 3.2. Modelling of Communications Demands

For the coverage area of a single RSU in Figure 1, which is located in the center of the 3-lane dual carriageway, with the central two lanes dedicated for trams, the number of vehicles is$\;N_{veh,t_{i}}\;$at time $t_{i}$. As a matter of fact, for the mixed traffic flow on the road, the vehicles do not always have MEC demands, and the vehicles are thus separated into five categories according to the need for the MEC. For the autonomous vehicles (without MEC) and normal vehicles (without MEC), they are not connected to the SDN RSU cloud, and do not ask for the communication and computing resources of the SDN RSU cloud. It is apparent to draw out the conclusion that the amount of the autonomous vehicles (without MEC) is small, since most of the autonomous vehicles are constantly capturing video and radar data, which are involved with the SDN RSU cloud. The number of the normal vehicles (without MEC) could be large, and it makes sense, since the normal vehicles are usually not equipped with many sensors, which are not always generating data flow. Before modeling the communication demands, the notations are presented in Table 1, pending the development of a communication requirements model.

From what has been described in Table 1, for the RSUs in the SDN RSU cloud, the fixed power $P_{R}$ for the RSU is utilized to transmit messages. Correspondingly, the transmit power of the vehicle is $P_{V}$. For the two-way mixed traffic flows, the number of vehicles in the coverage area of the RSU is represented by $N_{in}$, and the corresponding number of vehicles outside the coverage area of the RSU is represented by $N_{out}$. In the process of data dissemination, the interference of the relaying signal between the two opposing mixed traffic flows is relatively weak and initially assumed to be negligible [17]. What is more, for the simplicity of the communication demands model, the Doppler frequency shift effect is not taken into account. For the notations, the symbol ⎡X⎤ rounds the decimal number X to the next largest integer; for example, ⎡6.5⎤=7. According to the Shannon capacity equation, the maximum achievable transmission rate C(t) of a channel (whether V2I or V2V channel) is equal to the instantaneous (time-varying) capacity as follows:

(1) $$DataRate\left(t\right)=B_{w}\cdot \log\left(1+\frac{P\cdot {\left[L\right]}^{-\gamma }}{N_{0}\cdot B_{w}}\right)$$

As shown in Equation (1), the value of P is $P_{R}$ or $P_{V}$, according to the V2I or V2V channels. Moreover, L is given by $L_{n,i}$, when it comes to the circumstance of the V2I channel, and given by $L_{i,i+1}$, if otherwise.

In Figure 1, there are five types of vehicles on the lanes, all of which are covered in the SDN RSU cloud, assuming that it is composed of M RSUs. For the mixed traffic flow on the road, the overall communication demand $D_{t_{i\;}}$ for the SDN RSU cloud can be calculated by the following Equation (2):

(2) $$\{\begin{array}{c} D_{t_{i}}=\overset{M}{\underset{n=0}{\sum }}\overset{5}{\underset{m=1}{\sum }}\overset{N_{veh,t_{i}}-1}{\underset{j=0}{\sum }}demand_{n,m,j}\\ s.t.\;\;demand_{n,m,j}\leq DataRate\left(t\right) \end{array}$$

where $demand_{n,m,j}\;$refers to the single demand for the referring type of vehicle $Veh_{j}\;$in current time, and there are five categories of vehicles with M zones covered by M RSUs in the SDN RSU cloud. Therefore, for the vehicles in the area covered by the same RSU, the demands can be calculated by aggregating the five different categories of vehicles’ communication demands. Since there are two categories of vehicles, the autonomous vehicles (without MEC) and normal vehicles (without MEC), that are not involved in the communication of the SDN RSU cloud, the communication demands are from the remaining three categories of vehicles. For the autonomous vehicles (with MEC), their information flow usually has high quality requirements of delay. However, for the normal vehicles (with MEC), their data flow usually has relatively low requirements of delay. However, when it comes to the ITS applications, it is essential to meet the requirements of all information flows for the ITS system in the SDN RSU cloud. Moreover, in the experiment section, the delay requirements must be set as the minimum value of the various ITS application requirements.

### 3.3. SDN RSU Cloud

**Figure 2:**
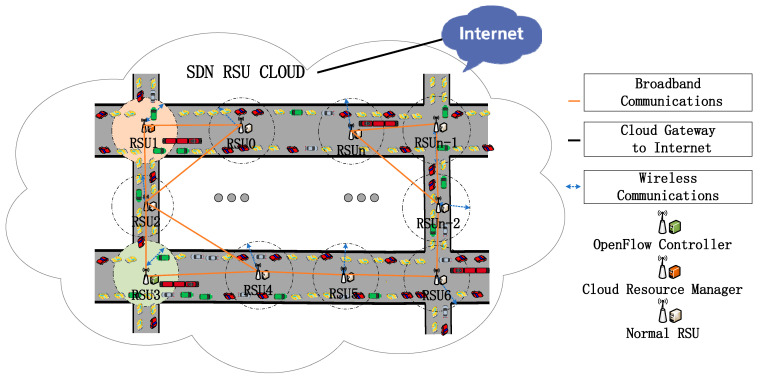
Schemes of the SDN RSU cloud.

After the modeling of communication demand for the mixed traffic flow, it is time to establish the mechanism of the SDN RSU cloud. In the proposed SDN RSU cloud, RSU plays three different roles: the OpenFlow Controller, the RSU Cloud Resource Manager (CRM), and the normal RSU functions. In the wireless field, self-organizing networks can also use SDN technology to implement SDN under dynamic topology [24]. The data exchange tool proposed for virtual machines and connected RSUs is the database of servers that store and compute data and switches that can exchange information from vehicles and connected RSUs. The RSU’s physical machines have enabled virtualization, and its virtual machines can run multiple replications on an individual machine. The switches on the RSUs are all OpenFlow switches, and perform the corresponding functions of the data forwarding layer. The proposed SDN RSU cloud mechanism is shown in Figure 2.

As shown in Figure 2, there are two additional RSU function characters in total, the OpenFlow Controller and RSU Cloud Resource Manager, which are based on the normal RSU functions. OpenFlow Controllers provide the flow table, which can update the system and manage the forwarding strategy. The CRMs distribute data in terms of hosting, service migration, data flow changes, and VM instantiating and(or) eliminating via the data plane; our design characters are simplified compared to the research done by Salahuddin et al. [25]. It is important to note that the RSU Cloud Resource Managers communicate with the OpenFlow Controllers via the data plane. In the designed cloud, the RSUs are distributed along the arterial and secondary roads of the whole area. In this case, the demands of each RSU are probably different, leading to unevenly distributed network demands, which would cause some RSUs to be too busy working with a heavy burden, while others idle with little burden. It is then time to manage the computing and communication resources of each RSU in the SDN RSU cloud for optimizing the network efficiency. The RSUs in the cloud not only transmit ITS service information, but they also serve as different characters according to the controller’s information given by the network manager. Those connections among the RSUs in Figure 2 are all wired broadband connections, thus ensuring the reliability of the data transmission. As a matter of fact, RSUs are mostly normal characters, which are not bestowed with the function of OpenFlow Controllers or Cloud Resource Managers.

### 3.4. Problem Analysis and Definition

#### 3.4.1. Model for Network Delay

The mathematical problem is then abstracted as follows, which gives the widely seen traffic networks fixed with a group of RSUs fluctuating communication demands. We assume that a network graph G=(V,E) is given with a fixed number of RSUs, whose V, *E* are representing the RSUs and the wired connections among the RSUs, respectively. For the traffic information services set S, the average demands set $D=\left\{D_{T_{0}},D_{T_{1}},D_{T_{2}}\ldots ,D_{T_{N}}\right\}$ that vary on time periods of $T=\left\{T_{0},T_{1},T_{2},\ldots T_{N}\right\}$. There is an initial configuration $W_{T_{0}}$ at time period $T_{0}$. For any edge, there is a bandwidth capacity $C_{E}$. For $T_{i}$, there is a demand $B_{M,K}$ at node M for service K with the average demand $D_{T_{i}}$. The mathematical model is designed to compute the best network configuration while minimizing the host numbers and RSU cloud delay for $T_{i}$, under the assumption that some service resource redundancy can be made possible.

The value $d_{M,n}$ is defined as the sum of the delays in all edges for the specific single routing path traversing from RSU M to n, which contains processing time $T_{p}$, queuing time$\;T_{Q}$, transmission time $T_{T}$, and propagation time $T_{Pr}$. Meanwhile, the processing time $T_{p}$ is usually a constant, which here is set as 10 μs. For a data packet’s queuing process, the Poisson distribution is used for modelling. Here, we assume that the Poisson process is modelled for the data packets’ queueing process with the packet interarrival rate λ and packet processing rate μ, whose mean and standard deviation are $\lambda_{M},\sigma_{M}$ and $\lambda_{S},\sigma_{S}$, respectively. We define the coefficient $Cf_{M}=\sigma_{M}/\lambda_{M}$, and $Cf_{S}=\sigma_{S}/\lambda_{S}$, as used by the Kingsman Formula to describe queuing delay $T_{Q}$ [26]:

Let L denote the transmission distance. According to science, the propagation speed of the signal in the wire is about 2/3 the light speed $v_{C}$. Meanwhile, the transmission time $T_{T}$ is calculated by the size of the packet $S_{P}$ divided by the Capacity $C_{E}$.

(3) $$T_{Pr}=\frac{L}{\frac{2}{3}\cdot v_{C}}$$

(4) $$T_{T}=\frac{S_{P}}{C_{E}}$$

What is more, the lookup table is generated based on the granularity g to describe the delay, which reflects the performance of the network.

#### 3.4.2. Mixed Integer Linear Programming Formulations

In the proposed model, the deep programmability of the SDN is employed to dynamically reconfigure the network configurations. The output parameters of the model are listed in Table 2.

Once the parameters of the model have been given (see Table 1 and Table 2), a joint optimization model can be established to find the optimal configuration that meets the latency requirements, while minimizing the number of hosts with energy restrictions. To work out the SDN cloud problem with energy restriction, we considered two weighted terms to turn the problem into two general sub-problems: the minimization of the delay problem, based on a given bandwidth to optimize the delay between RSUs and users with energy restriction; and the minimization of the service hosts’ number problem, which aims to deploy the minimum number of service hosts with energy restriction to maximize the entire network’s service capabilities. The truth is that all of these things are done under the assumption that there is an absolute necessity of redundancy in a short period, in order to prevent network instability caused by sudden communication demand surges. The object function of the problem is formulated in Equation (5).

(5) $$min\left\{\overset{N-1}{\underset{M=0}{\sum }}\overset{N-1}{\underset{n=0}{\sum }}\overset{{℧}_{M,n}-1}{\underset{J=0}{\sum }}B\cdot d_{M,n,J}+\overset{N-1}{\underset{M=0}{\sum }}\overset{S-1}{\underset{K=0}{\sum }}\left(1-B\right)\cdot Q_{M,K}\cdot R\right\}$$

Achieving the goal of a minimum number of hosts is inconsistent with minimizing the cloud delay. The constant B is therefore used to control the weight of the priority for Equation (5). When B is larger than 0.5, it means that delay takes precedence over the host number, and vice versa. Furthermore, the constraints of objective (5) are listed from (6) to (24) as follows:

(6) $$A\cdot X_{M,K}\geq Q_{M,K}-q_{M,K\;}\forall 0\leq M<N,0\leq K<S$$

(7) $$A\cdot (1-X_{M,K})+Q_{M,K}-q_{M,K\;}\geq 0\;\forall 0\leq M<N,0\leq K<S$$

The definition of the variable $X_{M,K}$ is restrained by (6) and (7), where if $Q_{M,K}-q_{M,K\;}>0$, $X_{M,K}$ equals 1 or otherwise equals 0.

(8) $$\underset{M,J}{\sum }w_{M,n,J}^{K}\geq B_{n,K}\;\forall 0\leq M,n<N,0\leq K<S,0\leq J<{℧}_{M,n}$$

(9) $$A\cdot Q_{M,K}\cdot F_{n,K}\geq \underset{J}{\sum }w_{M,n,J}^{K}\;\forall 0\leq M,n<N,0\leq K<S,0\leq J<{℧}_{M,n}$$

(10) $$Q_{M,K}\cdot F_{n,K}\leq \underset{J}{\sum }w_{M,n,J}^{K}\;\forall 0\leq M,n<N,0\leq K<S,0\leq J<{℧}_{M,n}$$

(11) $$w_{M,n,J}^{K}\leq \;A\cdot V_{M,n,J}^{K}\;\forall 0\leq M,n<N,0\leq K<S,0\leq J<{℧}_{M,n}$$

(12) $$w_{M,n,J}^{K}\geq \;V_{M,n,J}^{K}\forall 0\leq M,n<N,0\leq K<S,0\leq J<{℧}_{M,n}$$

The communication process could be summarized as such: if a demand occurs, there must be a load. The RSU servers must be able to meet or exceed the vehicle requirements listed below (8). At the same time, (9) and (10) impose restrictions on routes that allow the unrouted transmission of network loads only between RSU servers and users. Further, constraints are listed in (11) and (12) to ensure that there is sufficient load to carry the rules of the control plane, which is the natural cost of the system itself.

(13) $$l_{E}=\underset{M,n,J,K}{\sum }f_{M,n,J}^{E}\cdot w_{M,n,J}^{K}\;\forall 0\leq E<\left|E\right|$$

(14) $$l_{E}=g\cdot u_{E}\;\forall 0\leq E<\left|E\right|$$

(15) $$u_{E}=\overset{\left(C_{E}-1\right)/g}{\underset{i=0}{\sum }}i\cdot y_{E,i}\;\forall 0\leq E<\left|E\right|$$

(16) $$\overset{\left(C_{E}-1\right)/g}{\underset{i=0}{\sum }}y_{E,i}=1\;\;\forall 0\leq E<\left|E\right|$$

(17) $$d_{E}=\overset{\left(C_{E}-1\right)/g}{\underset{i=0}{\sum }}y_{E,i}\cdot u_{i,E}\;\;\forall 0\leq E<\left|E\right|$$

Constraint (13) describes the flow traverses between the RSU M and n as well as the load on the boundary, all of which contain the traffic generated and forwarded along the route. The delay on the edge E matches the load on it, and the index is used in the lookup table (LUT) to find it. Constraint (14) indicates that the load on the edge E is mapped to a group of g. Constraint (15) ensures that the traffic load at the edge does not exceed its capacity. At the same time, (16) and (17) make sure that only one specific value is used for the traffic load on the edge E and the delay associated with it.

(18) $$d_{M,n,J}=\overset{\left|E\right|-1}{\underset{E=0}{\sum }}f_{M,n,J}^{E}\cdot d_{E}\;\;\forall 0\leq M,n,M\neq n<N,\;0\leq J<{℧}_{M,n}$$

(19) $$d_{M,n,J}\cdot Q_{M,K}\cdot F_{n,K}\leq O_{K}\;\forall 0\leq M,n,M\neq n<N,0\leq K<S,\;0\leq J<{℧}_{M,n}$$

(20) $$d_{M,n,J}\cdot Q_{M,K}\leq d_{M,n,J}\;\;\forall 0\leq M,n,M\neq n<N,0\leq K<S,\;0\leq J<{℧}_{M,n}$$

Going another step further, the delay of the specific route between RSU M and n equals the summation of all the delays on its edges, which is shown in (18). Constraint (19) represents the delay threshold for service K, while constraint (20) explains if a routing path is being used.

(21) $$\overset{N-1}{\underset{M=0}{\sum }}Q_{M,K}\geq 1\;\forall 0\leq M<N,0\leq K<S$$

(22) $$\overset{N-1}{\underset{M=0}{\sum }}Q_{M,K}\leq H_{K}\;\;\forall 0\leq M<N,0\leq K<S$$

Constraint (21) guarantees that at least one host is working on the RSU M. On the other side, constraint (22) describes service K’s threshold on the number of service hosts. Salahuddin et al. supposed that all RSUs have infinite resources to satisfy any service’s demand, for the sake of simplicity [25]. However, it would be impossible to create RSUs without resource restrictions. To help solve this problem, the number of hosts for a single RSU is limited.

(23) $$\overset{S-1}{\underset{K=0}{\sum }}Q_{M,K}\leq H_{M}\;\;\forall 0\leq M<N,0\leq K<S$$

Formula (23) presents the resource constraints. For a single server of an RSU, it is reflected in constraint (23) that the maximum number of hosts is limited. This is also due to the fact that the financial service hosting cost produced by the internet service provider is proportional to the number of hosts. Therefore, in order to save on operating costs for the SDN RSU cloud, the number of hosts is strictly restrained.

(24) $$\overset{S-1}{\underset{K=0}{\sum }}Q_{M,K}\cdot \Delta \leq \Delta_{max}\;\;\forall 0\leq M<N,0\leq K<S$$

Constraint (24) is set to seek for a sustainable environment; the SDN RSU cloud is not only for extremely low latency, but also for the pursuit of energy conservation and environmental friendliness. Therefore, for a single RSU, the maximum energy consumption constraint is set to reduce carbon emissions and pursue environmentally sustainable development.

## 4. Results

### 4.1. Three Algorithms and Network Topology

OpenFlow switches control all traffic in the network through multiple paths to minimize the delay in the implementation of the SDN RSU cloud. Taken further, $d_{M,n,J}$ is a continuous variable greater than or equal to 0, while $Q_{M,K}$ is an integer variable. This model results in a mixed integer linear programming problem (MILP). Meanwhile, Gurobi is mix-programmed with Python for the working out of the MILP.

**Figure 3:**
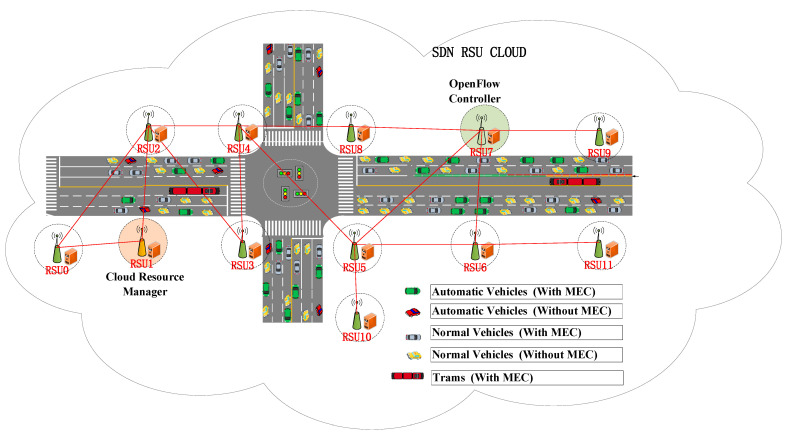
Detailed RSU cloud topology.

The proposed joint optimization algorithm hypothesizes that the computing and transmission resources of RSUs are finite with energy restriction, and here it is designated in the abbreviation form JF, which means Joint optimization algorithm with Finite resources. In comparison to the JF algorithm, there are another two optimization algorithms: (1) Host optimization (H), which hosts the service in the best manner possible to save on the number of hosts for the network demand with energy restriction; and (2) Delay optimization (D), which seamlessly hosts the best service to minimize delay with energy restriction, regardless of the number of host services. For JF, in order to test the effect of the different weight assignments between the delay and host number, three different assignments are set. That is, JF is set with the following hyperparameters: (DW = 0.3, HW = 0.7), (DW = 0.5, HW = 0.5), and (DW = 0.7, HW = 0.3). The research scenario locates in the coverage area of twelve fixed RSUs in the downtown area of the Longhua District in Shenzhen, China, where communication demands on the roads fluctuate during different time periods of traffic flows, and the detailed topology is shown in Figure 3 below.

From what can be seen in Figure 3, each RSU consists of an OpenFlow switch and a server, where the $RSU_{0}$ is composed of a server $h_{0}$ and a switch $s_{0}$, and is composed likewise for the rest of the RSUs. For the network topology in which the experiment has been conducted, each RSU communicates with five categories of vehicles within its range. Since each RSU is located in a different part of the city, the real-time transportation conditions and traffic flows are various, leading to the result that each RSU has different communication demands. Therefore, after the input of the communication demands and initial network configuration for the model, the JF algorithm has to solve the minimizing problem of the weighted communication delay and the number of hosts across the RSU SDN cloud with energy restriction. It is assumed that the paths from origin$\;RSU_{M}$ to destination $RSU_{n}$, which are represented in the lists, are acyclic elementary chains, where the labelled notations are from 0 to 11.

### 4.2. Experimental Environment and Initial Network Configurations

For the emulation environment, a microcomputer with the following specifications— Processor Intel ^®^ Core ™ i5-6300 3.20 GHz, 8 GB of RAM running the Operating System Windows 10 64 bits—was used for experiments.

It should be noted in advance that, for the intuitive and quantitative display of the results analysis, before the start of the experimental simulation, we aggregated the network demands generated by the mixed traffic flow that consisted of five categories of vehicles into the parameter D, which is listed in Table 3. With the input parameters all set as shown in Table 3, the packet size was 1240 bytes when working out on the experiment and numerical analysis.

As shown in Table 3, for various network demands, the number of services starts from *S* = 1 and increases at intervals of 1 until there is no feasible solution. The reality is that the greater the network demand, the smaller the number of services that can be run simultaneously. When the network demand is 5 Mbps, the number of services can be run is at most 22. When the network demand is greater than or equal to 50 Mbps, only one service can be run. For the network demand, it starts from 5 Mbps and increases at intervals of 5 Mbps. When it reaches 50 Mbps, since the maximum network demand with a feasible solution is 53 Mbps, the network demand increase interval at this time was changed to 1 Mbps. For a single network edge, the network capacity is 100 Mbps. The maximum number of hosts that can run for a single service is 5, and for a single RSU, the maximum number of hosts that can run is specified to be 5. The parameter g, which is used to control the graininess of the network, was set to 1. For the initial configuration of the network, it was assumed that the network had no working hosts running at the beginning. The control planes of the whole network were all initially set to 0. The parameter A, which is used for describing the constraints of the model, is a large constant and was set to 100000 here. What is more, in order to simplify the expression, for the JF algorithm, the “Delay Weight” was abbreviated as DW, and the “Host Weight” was abbreviated as HW. Meanwhile, the parameter B, which is utilized for controlling the “Delay Weight” (DW) and “Host Weight” (HW), was set to 0.3, 0.5, and 0.7, corresponding to JF (DW = 0.3, HW = 0.7), JF (DW = 0.5, HW = 0.5), and JF (DW = 0.7, HW = 0.3), respectively. The redundancy coefficient R was set to 1.5, which is set to prevent the surging increase of the network demand. To achieve the environmental sustainability, $\Delta_{max}$ was set to 10Δ for a single RSU, which represents the energy constraint for the whole SDN RSU cloud.

### 4.3. Service Number

**Figure 4:**
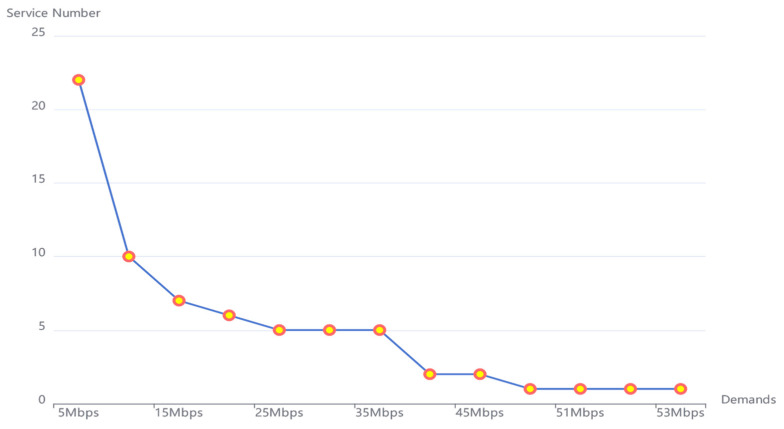
Max service number under various communication demands.

For the three algorithms (JF, H and D), in order to test out the maximum number of services that could run simultaneously for different communication demands, starting from *S* = 1, we increased the number of services at intervals of 1 until there was no feasible solution. Consequences show that, if given same communication demands, the maximum service number of the three algorithms (JF, H and D) are all the same. Experimental results showed that for smaller demands, more service numbers could be run simultaneously for the SDN RSU cloud. The detailed experimental results are shown in Figure 4 below.

It can be seen from Figure 4 that 22 service numbers can be run simultaneously for 5 Mbps, while only 1 service can be run when communication demands D ≥ 50 Mbps. This is consistent with the intuitive point of view that, when the network demand increases, though the goal is to achieve a balanced network load sharing on each path, too many services make it hard to obtain a feasible network configuration. At this time, when the service number increases, the network bottleneck often comes out, due to the constraints that certain paths need to meet, which makes it impossible to obtain a feasible solution for the SDN RSU cloud with energy restrictions. To further work out the impact of service number *S* on the overall SDN RSU cloud for JF, H and D, we conducted several experiments with different *S* for testing the impacts on cloud delay and the number of hosts, starting at 5 Mbps and growing in intervals of 5 Mbps. What is more, feasible solutions are with a maximum communication demand of 53 Mbps, and thus we further tested the conditions that demands were set at 50 Mbps, 51 Mbps, 52 Mbps and 53 Mbps. In our previous study [27], with 10 RSUs for the SDN RSU cloud topology, the maximum communication demand with a feasible solution was 97 Mbps. In the former research, though with different focuses on the research issue, to some extent it still showed that the network topology size and the number of RSUs had important effects on the existence of feasible solutions. That is, the larger the size of the SDN cloud network is, the harder it is to work out a feasible network configuration solution. Therefore, when designing an SDN RSU cloud, the size of the cloud should be controlled by a small size. When it comes to an SDN RSU cloud with a large network topology, grid division should be performed first. With each partition in the grid working well, the entire network can then work as a whole at the best network configuration.

During the process of the experiments, for all network demands, as long as the number of services did not exceed 10, it was found that the solution time of JF did not exceed one minute, so it had good real-time performance. As a matter of fact, when the number of services was increasing, the time for working out the feasible solution was also increasing. Taking 5 Mbps as an example, when *S* > 15, the time was longer than 1000 s and even exceeded 10,000 s when *S* = 22. Based on the real-time requirements for the SDN RSU cloud, it is usually unacceptable to have a time-consuming solution. But 5 Mbps can be seen as a special case, because it can run up to 22 services simultaneously; even for its adjacent circumstance at 10 Mbps, the maximum number of services that can run at the same time is only ten. The 5 Mbps demand can be considered as the circumstance that corresponds to the time of midnight, where mixed traffic flow consisting of 5 categories of vehicles is at a low volume on the road. Since the number of vehicles on the road is small at midnight, service numbers exceeding 10 services would generally not form. Furthermore, at 5 Mbps, even if it is assumed that the number of services will exceed 10, such as at the max service number of 22, the feasible solution would take more than 10,000 s to solve. But for this circumstance, the traffic flow is usually less likely to experience sudden surges or decreases, in which case the algorithm JF can still be considered adaptable.

As mentioned above, 5 Mbps is a special case for the SDN RSU cloud. For the representative analysis of experimental results, the network demand 10 Mbps was chosen, since the max service number is 10, which makes the model contain more instances for reference. In the preliminary experiment, various numbers of services were set and experimented from 1 to 10 for multiple times, until there was no feasible solution. All the consequences of the three algorithms (JF, D and H) are shown in Table 4.

From what has been shown in Table 4, the host’s number rose as the number of services changed from 1 to 10 for JF, D, and H. First of all, when comparing the impact of the three different hyperparameters configurations of the JF algorithm, generally, whether for cloud delay or the number of hosts, the results under the three different configurations were very close. Among them, for JF (DW = 0.3, HW = 0.7) and JF (DW = 0.5, HW = 0.5), the results of the two configurations were exactly the same, which indicates that there is no essential difference between the two configurations. When a higher weight is given to the cloud delay, things start to become different. For JF (DW = 0.7, HW = 0.3), i.e., giving a higher weight to the cloud delay, even when *S* = 1, 2, 3, 7, 10, there was no difference with the previous two hyperparameter configurations; when *S* = 4, 5, 6, 8, 9, the number of hosts was larger than the previous two configurations, but with a very small cloud delay reduction. This shows that blindly pursuing the reduction of cloud delay brings additional costs for the number of hosts, which is not worth it for the whole SDN RSU cloud.

### 4.4. Cloud Delay

**Figure 5:**
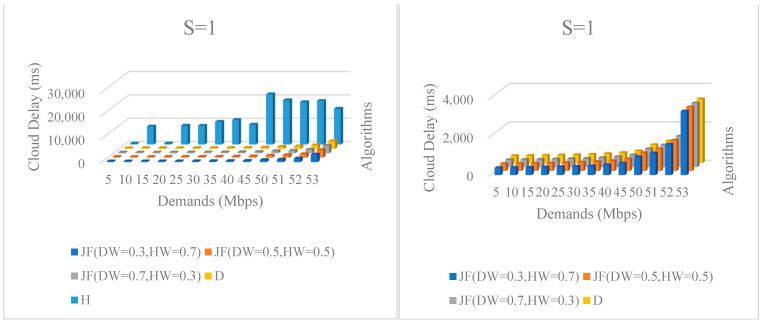
Cloud delay under various communication demands. The **left** part contains all three algorithms (JF with three different hyperparameters, D and H). The **right** removes the consequences of H algorithm for the convenience of comparison.

When comparing the D algorithm with the JF algorithm, which has three different hyperparameters configurations, it is obvious that, for the vast majority of *S* instances, the JF algorithm can achieve a network delay close to the D algorithm, and it can also reduce the number of hosts. Therefore, the JF algorithm is a better algorithm that takes both the delay and the number of hosts into account. Further observation and comparison of the H, JF and D algorithms shows that, though H algorithm has a much smaller number of working hosts for each *S* instance, the delay for the entire SDN RSU cloud increased explosively. What is more, the delay of the H algorithm increased by an order of magnitude when compared with the JF and D algorithms; therefore, an H algorithm that completely sacrifices the quality of network delay is obviously not desirable for the SDN RSU cloud. For the global comparison of all three algorithms’ cloud delay consequences under all demands, since feasible solutions only exist in S = 1 instance when demands D ≥ 50 Mbps, the *S* = 1 instance was taken for analysis and is shown in Figure 5.

Since the H algorithm pursues the minimum number of working hosts, the cloud delay of H was far larger than the other two algorithms. From the left part of Figure 5, for all three algorithms (JF with three different hyperparameters, D and H), it can be observed that when it came to the H algorithm, the cloud delay rose at an explosive speed, and reached over 5000 ms under all demands. From the rough appearance of the left part, as for cloud delay, it clearly shows that all three hyperparameters configurations of JF were approaching the consequences of the D algorithm, which means that the JF algorithm could ensure a good performance in the case of cloud delay while ensuring a smaller number of hosts. For the convenience of comparison, we removed the consequences of H in the right part of Figure 5. For the JF algorithm, it can be observed that, when it came to the circumstance of JF (DW = 0.3, HW = 0.7) and JF (DW = 0.5, HW = 0.5), nearly all the cloud delays were a little bit larger than the values of the D algorithm. Meanwhile, when it came to the case of JF (DW = 0.7, HW = 0.3), the sum of cloud delays for the D and JF algorithms were almost the same. This is because the JF methods focused on the optimization of delays, rather than the number of hosts, in this case.

### 4.5. Environmental Impact Analysis

#### 4.5.1. Number of Working Hosts

**Figure 6:**
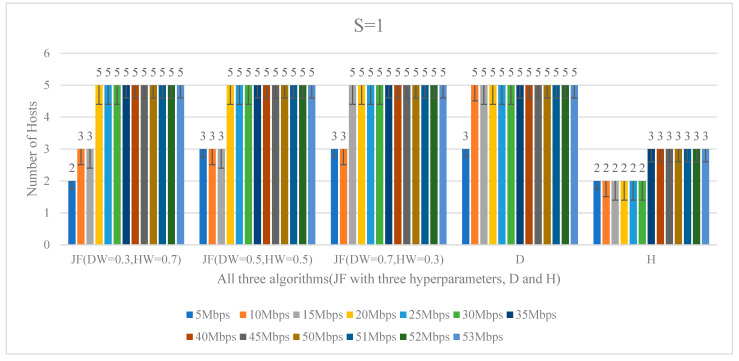
Hosts number under various communication demands for all three algorithms (JF with three different hyperparameters, D and H).

Therefore, in summary, the JF algorithm managed a good balance between the delay and the number of working hosts, and provided a good solution for the entire SDN RSU cloud. Furthermore, when the network demand reached 53 Mbps, it can be observed that the sum of cloud delays rose at an explosive speed, due to the reason that the entire networks were overloaded. Generally speaking, for all algorithms, it can be easily seen that the sum of cloud delays was arising as the demands increased, which is in accordance with the normal recognition. For a further detailed analysis of the hosts number, all the consequences of the three algorithms (JF with three different hyperparameters, D and H) are listed in Figure 6.

From what has been depicted in Figure 6, the hosts numbers were on the upward trend as the demands changed from 5 to 53 Mbps for all three optimization algorithms. What is more, from the visual perspective of Figure 6, the values of the left part, which contain JF (DW = 0.3, HW = 0.7), JF (DW = 0.5, HW = 0.5), JF (DW = 0.7, HW = 0.3) and D algorithms, were larger than the H algorithm in the right part. This was due to the restriction of delays, along with hosts number for the JF algorithm, and the pursuit of the minimum cloud delay for the D algorithm; the host numbers of the JF and D algorithms were equal to or larger than the H algorithm under the same demands.

Furthermore, when combining Figure 5 and Figure 6 together, it is intuitional to find that the algorithm JF (DW = 0.3, HW = 0.7), JF (DW = 0.5, HW = 0.5), compared with the D algorithm, usually had a slightly larger amount of delay, and generally had a relatively lower number of working hosts. Meanwhile, this did not rule out the case of the same number of working hosts, where the delays of the two were the same. But for most of the cases, the delays of the JF (DW = 0.7, HW = 0.3) and D algorithm were very close to the number of working hosts, which means that the JF (DW = 0.7, HW = 0.3) algorithm gave a higher priority for minimizing the cloud delay. In addition, when compared with the JF and D algorithms, though the number of working hosts of the H algorithm had been greatly reduced, this often brought about the surging increase in cloud delay, which was more apparent when the network demands grew greater. It is not worth making the cloud delay increase drastically for the purpose of reducing the working hosts number, as such a solution is unreasonable for the SDN RSU cloud.

#### 4.5.2. Energy Consumptions

It can be assumed that the energy consumption of a single working host is a fixed value Δ, when under the same communication demands. In [28], the number of CPU cycles required by a task was 40 M times, and one CPU cycle consumed 8.2 nJ, where the energy consumption was proportional to the size of the computing task. Based on the above conclusions, for the easier and more intuitional comparison of energy consumption of all three algorithms, the number of working hosts value can represent energy consumption under the same communication demands. The energy consumed by a working host in a time period $T_{N}$ can be regarded as a standard energy unit Δ. That is, taking 15 Mbps for the JF, D, and H algorithms as an example, the larger the value Δ is, the more energy would be consumed, all of which are as shown in the following Table 5. It is worth mentioning that in the optimization process of the objective function, a coefficient R was multiplied by the number of working hosts, which made some of the hosts run idly in order to prevent the surge in communication demands. The number of idling hosts should be removed for the analysis of the energy consumption, and thus the number of working hosts value would truly represent the energy consumption value under the same communication demands.

From what can be seen in Table 5, for 15 Mbps, JF (DW = 0.3, HW = 0.7), JF (DW = 0.5, HW = 0.5) had the same energy consumptions value Δ, which means these two versions had no essential difference. While for JF (DW = 0.7, HW = 0.3), it consumed more energy than JF (DW = 0.3, HW = 0.7) and JF (DW = 0.5, HW = 0.5), when *S* = 1,2,5,6, revealing that more attention was paid for reducing the cloud delay, rather than energy consumptions. What distinguished the D algorithm from the JF (DW = 0.5, HW = 0.5) was that it had more energy consumption when *S* = 4,6, and pursued extremely low cloud delays, regardless of anything else. Things become entirely different when it comes to H algorithm, and though it showed less energy consumption than the others, it came with an explosive increase in cloud delay. Although the H algorithm is inappropriate in most cases, it can still run for short-term system operation when energy restriction occurs.

## 5. Discussion

In the previous results analyses of Table 4 and Table 5, only the results of the single demand case were analyzed under the circumstances of different service numbers. In order to conduct a unified discussion with different communication demands, we analyzed the cases under 15 Mbps and 20 Mbps for comparison in Table 6, where the numbers of services were from one to six. What is extra distinctive from Table 5′s results is that the discussion here does not contain the full number of services, that is, *S* = 7 was removed. From the previous data in Figure 4, it can be seen that when the communication demand is 15 Mbps, the maximum number of services that can be run simultaneously is seven, and when the communication demand is 20 Mbps, the maximum number of services is six. Table 5 shows the energy consumption at 15 Mbps. Here, the delay and the number of working hosts of the SDN RSU cloud, as well as the energy consumption results, are placed in Table 6.

From what can be seen in Table 6, it is straightforward to find that, for 15 Mbps and 20 Mbps, regardless of the H algorithm results, JF (DW = 0.3, HW = 0.7) and JF (DW = 0.5, HW = 0.5) tended to have slightly larger delays and more working hosts with more energy consumptions when compared with the D algorithm, which is in accordance with the results analyses of Table 4 and Table 5. For example, in the case of 15 Mbps, when *S* = 1, 2, 4, 5, 6, Table 6 shows that the D algorithm usually had a smaller delay and a larger number of working hosts with slightly more energy consumption. Although the results of JF (DW = 0.7, HW = 0.3) were relatively close to the D algorithm, there were often the cases where the D algorithm increased the number of hosts with more energy consumptions in order to pursue a relatively slightly smaller delay. For example, when the communication demand was 20 Mbps with *S* = 2, the delay, the number of hosts, and energy consumptions of JF (DW = 0.7, HW = 0.3) were 434.16 ms, 11, and 7Δ, respectively, while those of the D algorithm were 434.02, 12, and 8Δ, respectively.

As mentioned earlier, the number of tasks needed for computing is proportional to the communication demand in a time period $T_{N}$. Therefore, for different communication demands of 15 Mbps and 20 Mbps, the energy consumptions need to be compared separately. Here, as shown in Table 6, in 20 Mbps, for JF (DW = 0.3, HW = 0.7) and JF (DW = 0.5, HW = 0.5), when *S* = 2,4, when compared to JF (DW = 0.7, HW = 0.3), had a slightly smaller number of working hosts and less standard energy consumptions, but with a slightly larger delays, showing that the weights of the three versions of the JF algorithm had different focuses. In terms of energy consumption, in general, it is also intuitional to know from the data corresponding to Table 6 that when the number of services and communication demands increase, the energy consumptions increase. Generally, it can also be found from Table 6 that for the same number of services cases, when the communication demands increase, the delay and the number of working hosts tend to increase correspondingly. It further indicates that the settings of the parameters also have an impact on the results of the delay and the number of working hosts. The results of JF (DW = 0.3, HW = 0.7) and JF (DW = 0.5, HW = 0.5) were often relatively close, when compared with JF (DW = 0.7, HW = 0.3). The latter usually endured a larger number of working hosts and more energy consumption, but with smaller delay than the former two algorithms. For the JF algorithm, multiple tuning instances of the DW and HW parameters are necessary. When it came to the global comparison of the JF, D, and the H algorithms, H pursued the extremely small number of working hosts with the least energy consumption, but completely ignored the explosive surge in delay, which is not desirable for the entire network.

## 6. Conclusions

In this article, we firstly introduced the ITS application scenario, which described how modeling the traffic demands of mixed traffic flows is an urgent issue, and contended that, to the best of our knowledge, there have been few efforts conducted for modeling the communication demands in detail by classifying vehicles into different categories. Furthermore, for the SDN RSU cloud mechanism, RSUs play three different roles: the OpenFlow Controller, the RSU Cloud Resource Manager, and the normal RSU functions. In addition, we designed an SDN data dissemination method for safer and greener transportation solutions to achieve the lowest overall SDN cloud delay with the least working hosts and fewest energy consumptions, which can be summarized as the mixed integer linear programming problem. The proposed software-defined networking data dissemination approach realized the interoperability of resource data in the SDN RSU cloud with energy restriction, which would find the optimal network configuration so that the whole network could achieve minimum delay and working host numbers with minimum energy consumptions. It can integrate similar or related resources through resource integration services to reduce the transmission of redundant data. On this basis, through resource data distribution planning services, according to the dynamic flow demands, under the condition of limited bandwidth, and in ensuring a certain amount of service resource redundancy, the resource allocation and balance of the RSU cloud can be realized.

The comparative experiments were designed to verify the dynamic resource management by comparing the joint optimization algorithm with three hyperparameters, that is, JF (DW = 0.3, HW = 0.7), JF (DW = 0.5, HW = 0.5), and JF (DW = 0.7, HW = 0.3), with D and H algorithms. The Delay optimization algorithm (D), seamlessly hosts the best service to minimize delay with energy restriction, without considering the number of hosting services; the Host optimization algorithm (H), regardless of the delay caused by the services, hosts the services in the best manner to save the number of hosts for meeting the network demand with energy restriction. Results showed that JF (DW = 0.3, HW = 0.7) and JF (DW = 0.5, HW = 0.5) approached the same objective optimization as algorithm D in the performance of cloud delays, with fewer working hosts and less energy consumption under some given demands. This means that, if given good hyperparameters, the JF algorithm usually performs better in the SDN RSU cloud, and has good applicability. In addition, while the number of working hosts and energy consumptions for H algorithms are significantly reduced, when compared to the JF and D algorithms, it tended to lead to a surge in cloud delay, whose phenomena was more apparent as network demands increased. A significant surge in cloud delay to reduce the number of hosts and energy consumptions is not worthy, and such a solution is not applicable for most cases of the SDN RSU cloud. Meanwhile, the proposed JF algorithm optimized the RSU cloud delay, while pursuing the least amount of working hosts and minimum energy consumptions, which also has significance for environmental protection and carbon emissions.

The analysis further shows that the number of RSUs in the SDN RSU cloud would have an important impact on the existence of feasible solutions. In other words, the larger the SDN cloud network topology, the harder it is to find a feasible network configuration. Therefore, when designing an SDN RSU cloud for greener future mobility of the intelligent transportation systems, the size of the cloud should be controlled. In terms of RSU access, future works can consider more complex scenarios, such as overlapping coverage of multiple RSUs and assisted driving for vehicles, for the mixed traffic flow. For further research directions, security and privacy issues bring the SDN RSU cloud more challenges, and it would be valuable to solve the network security issues. What is more, for future research of the SDN RSU cloud, deep reinforcement learning can be used to work out the problem of time-varying characteristics of the network demands, which will further improve the real-time performance.

## Figures and Tables

**Table 1:** Definitions for the communications demand notations.

Input Parameters	Descriptions
$L_{n,i}$	Distance between the nth RSU and ith vehicle.
$L_{i,i+1}$	Distance between the ith vehicle and i+1th vehicle.
$N_{in}$	Number of vehicles inside the area which is covered by the RSU.
$N_{out}$	Number of vehicles outside the RSU’s coverage area.
*B_w_*	Bandwidth used by the wireless communications channels between the vehicle and the RSU.
$N_{0}$	Power spectrum density of the white gaussian noise.
γ	Path loss exponent.
$P_{R}$	The transmission power of the RSUs.
$P_{V}$	The transmission power of the vehicles.
*S*	Service number of the traffic information set.
N	RSUs number.
$B_{M,K}$	The Demand at $RSU_{M}$ for service K, ∀0≤M<N,0≤K<S.
$F_{n,K}$	$=\{\begin{array}{c} 1,\;if\;a\;demand\;occurs\;on\;RSU_{n}\;for\;service\;K,\forall 0\leq n<N,0\leq K<S\\ 0,otherwise \end{array}$
${℧}_{M,n}$	Paths number for RSU M to RSU n.
$f_{M,n,J}^{E}$	$=\{\begin{array}{c} 1,\;if\;RSU_{M}\;to\;RSU_{n}\;uses\;path\;J\;with\;edge\;E,\forall 0\leq M,n<N,0\leq J<{℧}_{M,n},0\leq E\leq \left|E\right|.\\ 0,otherwise \end{array}$
*C_E_*	Bandwidth capacity of edge E.
$H_{K}$	Number of hosts’ threshold on Service K, 0≤K<S.
$O_{K}$	Delay threshold of service K,0≤K<S.
$H_{M}$	$RSU_{M}$’s threshold on the number of service hosts, ∀0≤M<N.
$C_{h_{M,K}}$	Traffic processing capacity of the single host, ∀0≤M<N,0≤K<S.
g	To control the granularity of the Lookup Table, $\forall 0\leq g<C_{E}$.
$u_{i,E}$	Load$\;i^{\prime }s\;$delay on edge E, $\forall 0\leq i<C_{E},0\leq E\leq \left|E\right|$.
$q_{M,K}$	$=\{\begin{array}{c} 1,\;if\;service\;K\;was\;hosted\;on\;RSU_{M}\;,\forall 0\leq M<N,0\leq K<S\\ 0,otherwise \end{array}$
$v_{M,n,J}^{K}$	$=\{\begin{array}{c} 1,\;if\;a\;control\;plane\;existed\;on\;path\;J\;from\;RSU\;M\;to\;n\;for\;service\;K,\\ \forall 0\leq M,n<N,0\leq K<S,0\leq J<{℧}_{M,n}\\ 0,otherwise \end{array}$
A	A large constant.
B	The weight of the priority for the equations, 0≤B≤1.
R	A constant, usually greater than or equal to 1. The parameter for ensuring a certain amount of service resource redundancy.
Δ	A constant, which is used to represent the standard energy consumption unit of a working host.
$\Delta_{max}$	$A\;\mathrm{constant},\;\mathrm{which}\;\mathrm{is}\;\mathrm{the}\;\max\;\mathrm{energy}\;\mathrm{constraint}\;\mathrm{for}\;\mathrm{an}\;\mathrm{RSU}\;\mathrm{in}\;a\;\mathrm{time}\;\mathrm{period}\;T_{N}$, represented in the form of standard energy consumption unit Δ.

**Table 2:** Definitions for the output Notations.

Output Parameters	Descriptions
$Q_{M,K}$	$=\{\begin{array}{c} 1,\;if\;service\;K\;now\;is\;hosted\;on\;RSU_{M}\;,\forall 0\leq M<N,0\leq K<S.\\ 0,otherwise \end{array}$
$w_{M,n,J}^{K}$	The load for service K on path *J* from $RSU_{M}$ to $RSU_{n}$, $\forall 0\leq M,n<N,0\leq K<S,0\leq J<{℧}_{M,n}.$
$V_{M,n,J}^{K}$	$=\{\begin{array}{c} 1,\;if\;a\;control\;plane\;exists\;on\;path\;J\;from\;RSU\;M\;to\;n\;for\;service\;K,\\ \forall 0\leq M,n<N,0\leq K<S,0\leq J<{℧}_{M,n}\\ 0,otherwise \end{array}$
$l_{E}$	The load on edge E, ∀0≤E≤|E|.
$d_{E}$	The delay on edge E, ∀0≤E≤|E|.
$u_{E}$	The load on edge *E* is mapped to a bunch of $u_{E}$.
$y_{E,i}$	$=\{\begin{array}{c} 1,\;if\;the\;load\;on\;edge\;E\;equals\;to\;i\cdot g,\forall 0\leq i<C_{E},0\leq E\leq \left|E\right|.\\ 0,otherwise \end{array}$
*d_M,n,J_*	The delay on path J from $RSU_{M}$ to $RSU_{n}$,$\forall 0\leq M,n<N,0\leq J<{℧}_{M,n}$.
$X_{M,K}$	$=\{\begin{array}{c} 1,\;if\;Q_{M,K}-q_{M,K\;}>0\;,\forall 0\leq M<N,0\leq K<S.\\ 0,otherwise \end{array}$

**Table 3:** Initial Network Configuration Parameters.

Parameter	Value
S	{1, 2, 3, 4, … 22}
N	12
D	{5 Mbps, 10 Mbps, 15 Mbps, 20 Mbps,25 Mbps, 30 Mbps, 35 Mbps, 40 Mbps, 45 Mbps, 50 Mbps, 51 Mbps, 52 Mbps, 53 Mbps}
$C_{E}$	100 Mbps
$H_{K}$	5, ∀0≤K<S
$O_{K}$	60 ms, ∀0≤K<S
$H_{M}$	5, ∀0≤M<N
g	1
$q_{M,K}$	0, ∀0≤M<N,0≤K<S
$v_{M,n,J}^{K}$	0, $\forall 0\leq M,n,M\neq n<N,0\leq K<S,\;0\leq J<{℧}_{M,n}$
A	100,000
B	0.3, 0.5, 0.7
R	1.5
$\Delta_{max}$	10Δ

**Table 4:** Consequences of all three algorithms when demand = 10 Mbps for all services from 1 to max.

10 Mbps	S	1	2	3	4	5	6	7	8	9	10
JF (DW =0.3, HW = 0.7)	Delay (ms)	388.54	399.48	407.41	420.92	422.02	464.49	541.98	582.26	580.33	1052.40
	Hosts	3	8	9	11	15	20	23	26	32	36
JF (DW =0.5, H_weight = 0.5)	Delay (ms)	388.54	399.48	407.41	420.92	422.02	464.49	541.98	582.26	580.33	1052.40
	Hosts	3	8	9	11	15	20	23	26	32	36
JF (DW =0.7, HW = 0.3)	Delay (ms)	388.54	399.48	407.41	419.55	419.86	463.55	541.98	579.47	578.90	1052.41
	Hosts	3	8	9	12	18	21	23	29	33	36
D	Delay (ms)	388.00	399.00	407.41	419.55	419.00	463.00	541.00	578.00	578.90	1052.40
	Hosts	5	9	9	12	20	23	29	32	33	36
H	Delay (ms)	7794.10	17,520.82	10,975.83	13,880.07	4933.95	11348.4	12,455.35	18,566.53	14,772.85	12,476.71
	H	2	3	5	6	8	9	11	12	14	20

The algorithm JF is given with three different hyperparameters for comparison, and service numbers are set from 1 to 10, since *S* = 11 makes the model have infeasible solutions.

**Table 5:** Energy consumption of all three algorithms when demand = 15 Mbps.

Demands = 15 Mbps	S	1	2	3	4	5	6	7
JF (DW = 0.3, HW = 0.7)	Energy Consumptions (Δ)	2	5	6	8	13	16	18
JF (DW = 0.5, HW = 0.5)	Energy Consumptions (Δ)	2	5	6	8	13	16	18
JF (DW = 0.7, HW = 0.3)	Energy Consumptions (Δ)	3	6	6	8	14	17	18
D	Energy Consumptions (Δ)	3	6	6	10	14	18	18
H	Energy Consumptions (Δ)	1	2	3	4	5	6	10

**Table 6:** Consequences of all three algorithms when demand = 15 Mbps, 20 Mbps for *S* = 1, 2, 3, 4, 5, 6.

Demands	Algorithms	S	1	2	3	4	5	6
15 Mbps	JF (DW = 0.3, HW = 0.7)	Delay (ms)	398.76	416.45	433.46	453.53	451.58	565.56
		Hosts	3	8	9	12	20	24
		Energy Consumptions (Δ)	2	5	6	8	13	16
	JF (DW = 0.5, HW = 0.5)	Delay (ms)	398.76	416.45	433.46	453.53	451.58	565.56
		Hosts	3	8	9	12	20	24
		Energy Consumptions (Δ)	2	5	6	8	13	16
	JF (DW = 0.7, HW =0.3)	Delay (ms)	397.56	415.04	433.46	453.53	450.61	564.35
		Hosts	5	9	9	12	21	26
		Energy Consumptions (Δ)	3	6	6	8	14	17
	D	Delay (ms)	397.56	415.04	433.46	452.95	450.61	563.98
		Hosts	5	9	9	15	21	27
		Energy Consumptions (Δ)	3	6	6	10	14	18
	H	Delay (ms)	486.72	7958.02	7652.37	6677.24	13,693.21	21,584.32
		Hosts	2	3	5	6	8	9
		Energy Consumptions (Δ)	1	2	3	4	5	6
20 Mbps	JF (DW = 0.3, HW =0.7)	Delay (ms)	409.98	435.57	472.37	500.14	494.15	851.65
		Hosts	5	9	9	15	21	24
		Energy Consumptions (Δ)	3	6	6	10	14	16
	JF (DW = 0.5, HW = 0.5)	Delay (ms)	409.98	435.57	472.37	500.14	494.15	851.65
		Hosts	5	9	9	15	21	24
		Energy Consumptions (Δ)	3	6	6	10	14	16
	JF (DW = 0.7, HW = 0.3)	Delay (ms)	409.98	434.16	472.37	498.86	494.15	851.65
		Hosts	5	11	9	17	21	24
		Energy Consumptions (Δ)	3	7	6	11	14	16
	D	Delay (ms)	409.98	434.02	471.89	498.86	494.15	851.65
		Hosts	5	12	11	17	21	24
		Energy Consumptions (Δ)	3	8	7	11	14	16
	H	Delay (ms)	8163.69	17,221.68	5189.11	26,009.75	25,154.44	13,208.18
		Hosts	2	3	5	6	8	14
		Energy Consumptions (Δ)	1	2	3	4	5	9

## Data Availability

The data presented in this research are available from the corresponding author upon request.
